# The effects of increasing longevity and changing incidence on lifetime risk differentials: A decomposition approach

**DOI:** 10.1371/journal.pone.0195307

**Published:** 2018-04-19

**Authors:** Marcus Ebeling, Karin Modig, Anders Ahlbom, Roland Rau

**Affiliations:** 1 University of Rostock, Chair of Demography, Rostock, Germany; 2 Max Planck Institute for Demographic Research, Rostock, Germany; 3 Karolinska Institutet, Institute of Environmental Medicine (IMM), Stockholm, Sweden; Scientific Institute of Public Health (WIV-ISP), BELGIUM

## Abstract

Increasing longevity can distort time trends in summary measures of health and mortality, such as the lifetime risk of getting diseased. If not observing a cohort, this lifetime risk is calculated with cross-sectional data on age-specific incidence and survival. In those instances, incidence and survival may work in opposite directions resulting in lifetime risk estimates where, reductions in incidence might be offset by a simultaneous longevity increase. The proposed method decomposes the difference between two lifetime risks into contributions of changing incidence and changing survival. The approach can be extended to measure the contributions of changes in disease related mortality and even case fatality. We illustrate the method with hypothetical examples as well as remaining lifetime risk at age 60 of experiencing a myocardial infarction, colorectal cancer and hip fractures for Swedish males. The empirical examples show that the influence of increasing longevity on the development of lifetime risk depends on the respective age profile of occurrence. In the cases of myocardial infarction and hip fracture, longevity increases of the general population counterbalanced or even exceeded the substantial gains in disease incidence, while for colorectal cancer, the lifetime risk was almost unaffected by the longevity improvement. This was because colorectal cancer has an on average earlier onset than myocardial infarction and hip fracture.

## Introduction

Lifetime risk expresses the probability to develop a certain disease throughout lifetime or from a certain age onward (remaining lifetime risk). This makes it a useful indicator for monitoring the burden of a disease in a population. It is, for instance, widely applied in cancer research [1–4].

There is no common procedure for the calculation of lifetime risk. In many studies, it rests on longitudinal data for cohorts, such as the Rotterdam study, the Framingham Heart Study or population register data [5, 6]. Lifetime risk in these approaches refers to observed lifetimes of individuals. However, lifetime risk has also been calculated based on cross-sectional data [1, 4, 7]. This approach is sometimes called “current probability method” [8]. In such a context, lifetime risk is based on observed disease and death patterns at a certain point in time. For at least two practical reasons, the use of cross-sectional lifetime risks are a valuable alternative to estimates based on longitudinal data. First, the data requirements are much lower for such a lifetime risk since no data with a sufficiently long follow-up time are necessary. Second, a cross-sectional lifetime risk summarizes the current burden in the population, whereas a lifetime risk based on longitudinal data summarizes past trends.

For the calculation of lifetime risk based on cross-sectional data, death and incidence rates for the respective period and population are required. Essentially by using standard life table techniques, these death and incidence rates are assumed to apply as if a real cohort would pass through time. The necessary assumption of time-invariant mortality and incidence are obviously false but since we are aiming to depict the current situation, this is, nevertheless, acceptable. Let us assume we have age-specific death and incidence rates of myocardial infarction for males aged 60 and older in a certain period. For this example, an interpretation of a lifetime risk would be: a 60 year-old male at the specific point in time has on average a remaining lifetime risk of, for instance, 30% to experience a myocardial infarction, if mortality and incidence stay constant over time.

The underlying risk population for a cross-sectional lifetime risk is usually drawn from a life table [1, 4, 8]. Accordingly, the two exit possibilities are the disease of interest and death as competing risk. The resulting lifetime risk estimate is based on the population age-structure of the life table, which can be regarded as a closed and stationary population, and which, solely depends on age-specific incidence and survival. Hence, differences in lifetime risk over time or between populations can arise from differences in incidence or in general survival. However, changes in just one of the two are unlikely in reality. In this instance, the interplay of survival and disease incidence can distort lifetime risk estimates and complicates meaningful comparisons of cross-sectional lifetime risks over time or between populations [1]. For example, a recent study has shown that the lifetime risk of hip fracture increased over time, despite declining age-specific incidence rates [7]. But how much of the change in lifetime risk can be attributed to each of the two factors?

In this paper, we aim to present a simple method to disentangle the two factors, allowing us to quantify how much of the change in lifetime risk is due to 1) changes in age-specific incidence and 2) changes in age-specific survival in the general population. We illustrate the method with an example based on artificial data and an application to empirical estimates of lifetime risk of myocardial infarction, hip fracture and colorectal cancer, using incidence and mortality of Swedish males.

## Methods

### Decomposing lifetime risk

Assuming that our incidence rates depict the first occurrence of the disease of interest, the rate of either dying or getting diagnosed at age x, *μ*_*x*_, can be written as

$$\begin{array}{c} \mu_{x}=m_{x}+I_{x} \end{array}$$ (1)

where *m*_*x*_ is the death rate at age x and *I*_*x*_ is the incidence rate of getting diseased at age [1, 9]. For these estimation, death rates ideally refer to the disease-free population because they are the exit rate for those who die before getting diseased, whereas incidence covers the exit rate for those who get diseased before they die. In empirical application, however, these data requirements can not always be fulfilled and alternative death rates must be used (see data and materials for further discussion). Note that death and incidence rates are referring to age intervals, which can also be bigger than one year of age. However, the length of the age-groups does not change the basic derivation of the decomposition method. To improve readability, we therefore excluded the notation of intervals throughout the manuscript. Moreover, the whole approach assumes constant rates within age intervals and also independence of age-specific death and incidence rates.

The probability of staying alive and healthy within one age interval x can be expressed by *exp*[−*μ*_*x*_]. Hence, the fraction alive and healthy at age x can be calculated by $exp[-\sum_{0\leq x_{i}<x}\mu_{x_{i}}]$, where *x*_*i*_ denotes the running index of the sum, which is age. The lifetime risk of becoming diseased from age x onward, *lr*_*x*_, can then be calculated by

$$\begin{array}{c} lr_{x}=\sum_{x\leq x_{i}\leq \omega }I_{x_{i}}\;exp\left[-\sum_{0\leq x_{i}<x}\mu_{x_{i}}\right], \end{array}$$ (2)

where *ω* denotes the highest age attained. Eq 2 can be rewritten as

$$\begin{array}{c} lr_{x}=\sum_{x\leq x_{i}\leq \omega }I_{x_{i}}\;exp\left[-\sum_{0\leq x_{i}<x}I_{x_{i}}\right]\;exp\left[-\sum_{0\leq x_{i}<x}m_{x_{i}}\right]. \end{array}$$ (3)

For simplicity, we will write $\phi_{x_{i}}$ for $I_{x_{i}}\;exp\left[-\sum_{0\leq x_{i}<x}I_{x_{i}}\right]$ and $l_{x_{i}}$ for $exp[-\sum_{0\leq x_{i}<x}m_{x_{i}}]$. Hence, Eq 3 changes to

$$\begin{array}{c} lr_{x}=\sum_{x\leq x_{i}\leq \omega }\phi_{x_{i}}l_{x_{i}}. \end{array}$$ (4)

We are interested in decomposing the change in lifetime risk to experience a given disease, denoted with Δ, between two time points A and B or, more generally, the difference between two populations:

$$\begin{array}{c} \Delta =lr_{x,A}-lr_{x,B}. \end{array}$$ (5)

The following methodological outline and derivation of the decomposition is based on previous studies, which provide general results for mathematical problems of such kind [10, 11]. Given the general definition of lifetime risk of Eq 4, we can rewrite Eq 5 to

$$\begin{array}{c} \Delta =\sum_{x\leq x_{i}\leq \omega }\phi_{x_{i},A}l_{x_{i},A}-\sum_{x\leq x_{i}\leq \omega }\phi_{x_{i},B}l_{x_{i},B}. \end{array}$$ (6)

Rearranging Eq 6 leads to

$$\begin{array}{c} \Delta =\underset{\text{Contribution}\;\text{of}\;\text{Changing}\;\text{Survival}\;\text{Conditions}}{\underset{︸}{\sum_{x\leq x_{i}\leq \omega }[l_{x_{i},A}-l_{x_{i},B}]\frac{\phi_{x_{i},A}+\phi_{x_{i},B}}{2}}}+\underset{\text{Contribution}\;\text{of}\;\text{Changes}\;\text{in}\;\text{Incidence}}{\underset{︸}{\sum_{x\leq x_{i}\leq \omega }[\phi_{x_{i},A}-\phi_{x_{i},B}]\frac{l_{x_{i},A}+l_{x_{i},B}}{2}}}. \end{array}$$ (7)

The omitted steps for deriving the expression in Eq 7 are listed in the S1 Appendix. Eq 7 provides now two distinct interpretable terms. The left term expresses the contribution of changing survival conditions to the difference in the lifetime risk between populations A and B. The right term expresses the contributions of changes in disease incidence to the difference in the lifetime risk between populations A and B. For both left and right term, age-specific differences in incidence and survival are standardized by average age-specific survival and incidence between both populations, respectively. This could be interpreted as if mortality or, respectively, incidence would have been the same for both populations. Note in the S2 Appendix you find an extension of this decomposition, which additionally includes the mortality of the specific disease.

### Hypothetical example

Table 1 presents three hypothetical examples to illustrate the decomposition as presented in Eq 7. In the first example (I), survival improves as reflected in the *l*_*x*_–columns, while the incidence proportions are unchanged. The lifetime risk rose by 12 percentage points from *lr*_*A*_ = 0.24 to *lr*_*B*_ = 0.36. Because we subtracted B from A we obtained a negative value. The contribution to the increase in the lifetime risk from changes in incidence proportions (last column) is obviously zero since *i*_*A*,*x*_ and *i*_*B*,*x*_ do not differ at any age. As expected, the difference in lifetime risk can be completely attributed to improvements in survival.

**Table 1:** Illustration of the decomposition method with hypothetical examples. **(I) Changes in age-specific survival**. **(II) Changes in age-specific incidence**. **(III) Changes in age-specific survival and incidence**. In all three examples, we decomposed the change from *A* to *B* (*A* − *B*).

						Contribution of Change in Age-Specific	
Scenario	Age *x*	*l*_*A*,*x*_	*l*_*B*,*x*_	*ϕ*_*A*,*x*_	*ϕ*_*B*,*x*_	Survival^†^	Incidence^‡^
(I) Change in Survival	1	1.0	1.0	0.0	0.0	0.00	0
	2	0.7	0.8	0.2	0.2	-0.02	0
	3	0.2	0.4	0.3	0.3	-0.06	0
	4	0.1	0.2	0.4	0.4	-0.04	0
*lr*_*A*_ = 0.24 *lr*_*B*_ = 0.36 Δ = −0.12					∑	-0.12	0
(II) Change in Incidence	1	1.0	1.0	0.0	0.0	0	0.00
	2	0.7	0.7	0.2	0.1	0	0.07
	3	0.2	0.2	0.3	0.4	0	-0.02
	4	0.1	0.1	0.4	0.2	0	0.02
*lr*_*A*_ = 0.24 *lr*_*B*_ = 0.17 Δ = 0.07					∑	0	0.07
(III) Changes in Survival **and** Incidence	1	1.0	1.0	0.1	0.2	0.000	-0.100
	2	0.5	0.8	0.2	0.1	-0.045	0.065
	3	0.3	0.4	0.3	0.4	-0.035	-0.035
	4	0.1	0.2	0.4	0.2	-0.030	0.030
*lr*_*A*_ = 0.33 *lr*_*B*_ = 0.48 Δ = −0.15					∑	-0.11	-0.04

^†^ Estimated by first part of Eq 7.

^‡^ Estimated by second part of Eq 7.

The complementary picture is provided by the second example (II). Age-specific survival does not differ between time points A and B. Instead, the age-specific incidence proportions changed over time. The overall reduction in lifetime risk of 0.07 is therefore equivalent to the sum of the contributions from the different age categories.

Although all examples are hypothetical, the third example (III) is probably closest to reality because contributions to lifetime risk differences originate from changes in age-specific survival as well as from changes in age-specific incidence proportions. The lifetime risk increased by 15 percentage points from 0.33 to 0.48. Our decomposition method allows us to disentangle the overall effect into contributions due to varying mortality conditions and varying age-specific incidence. It turns out that the increase in lifetime risk is due to a combination of higher incidence proportions and higher survival. In addition to this qualitative assessment, we can also state that improved survival contributed almost three times more (0.11) to the increase in lifetime risk than the actual incidence risk (0.04).

## Empirical examples

### Material and data

We applied the method to remaining lifetime risk of getting diagnosed with myocardial infarction, colorectal cancer and hip fracture for Swedish males at age 60. Note, however, that the decomposition can be applied to lifetime risk starting at any age. For the different diseases, we compared the lifetime risk between two different time points. These are 1987 and 1994 for colorectal cancer, 1994 and 2014 for hip fractures and 1994 and 2004 for myocardial infarction.

The incidence estimates for the three disease outcomes were obtained from Swedish registry data maintained by Statistics Sweden and the National Board of Health and Welfare [12]. For myocardial infarction and hip fracture, the first event occurring after age 60 after a seven year disease free period was identified from the National Patient Register. For colorectal cancer, the date of the diagnosis of the cancer was collected from the Swedish Cancer Register. The incidence counts have been smoothed across age and time to reduce random fluctuations, using the MortalitySmooth package in R [13, 14].

Death—the competing risk of getting diseased— should ideally be measured by the death rates of the disease-free population. This requires sufficiently detailed data sources, which allow to follow individuals over time, in order to determine whether they can be considered disease-free or not. In most cases, however, such detailed data are not available, and therefore, alternative data sources must be used. In our example, death rates are based on death counts and population data of the total Swedish male population, provided by the Human Mortality Database [15]. For two reasons, these death rates are very likely overestimating mortality in a cross-sectional setting. First, persons who get diseased and die in the same year are considered in the incidence as well as in the death rate and are therefore counted as two separate exits. Second, in our quasi-cohort perspective, death rates at consecutive ages are also potentially too high because total population death rates still contain the diseased population. It is very likely that this population suffers from higher mortality than the disease-free population. For practical reasons, however, the use of such death rates is acceptable because this misclassification likely overestimates mortality, and thus, leads to upper bound estimates of lifetime risk and the contribution of increased survival. Note that the bias of using such death rates depends on the respective relationship between mortality and the disease of interest.

**Fig 1:**
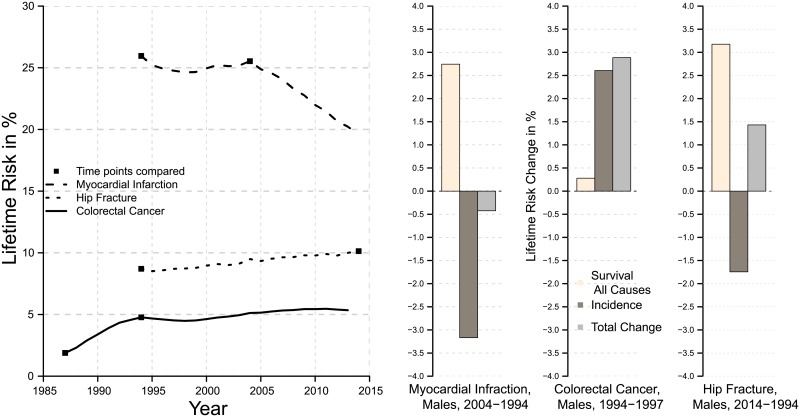
Remaining lifetime risk at age 60 and lifetime risk decomposition for myocardial infarction, hip fracture and colorectal cancer, Sweden, males.

The left panel of Fig 1 depicts the time trends of lifetime risk for the respective diseases. The dots mark the time points, which are selected for the decomposition. The selection is based on the pattern of the respective lifetime risks. Accordingly, for myocardial infarction, we choose the end points of a period with almost stagnating levels of lifetime risk. For hip fracture, both time points comprise a period of slight increases and for colorectal cancer, the time points are the boundaries of a period of increasing lifetime risk. The right panel illustrates the results of the decomposition. The bars show the contribution of changing survival and of changing incidence as well as the total change in lifetime risk.

### Results

In the case of myocardial infarction, the lifetime risks are almost similar at the two time points, which could lead to the conclusion that there have been no improvements in incidence above age 60. By applying the decomposition, however, it becomes clear that declining incidence would have generated a decrease in lifetime risk by more than three percentage points but increasing longevity prevented this decline. If only mortality would have changed between the two time points, the mortality improvements and, hence, the higher number of survivors to older ages would have resulted in an increase of lifetime risk by more than 2.5 percentage points. In sum, the counteracting factors resulted in an overall change of lifetime risk by less than a half percentage point.

For hip fracture, we observe a slight but steady increase in remaining lifetime risk above age 60 over time. This rise, however, is entirely driven by increasing longevity. The declining incidence, given the same mortality at both time points, would have contributed to a decrease of lifetime risk by more than 1.5 percentage points, whereas the survival improvements, given the same incidence at both time points, would have generated an increase by more than 3 percentage points. Accordingly, the total change sums up to an increase by almost 1.5 percentage points.

Colorectal cancer is an example, where lifetime risk increases relatively steeply in a short period of time. In contrast to the other two examples, incidence contributes to a rise in the remaining lifetime risk at age 60. Consequently, the incidence between 1987 and 1994 has increased. Rising incidence alone would have generated an increase of lifetime risk by more than 2.5 percentage points. Also the role of improving survival for changes in the lifetime risk are different compared to the other two examples. The on average earlier occurrence of colorectal cancer lowered the impact of the longevity advancement. Hence, the contribution of increasing survival, given the same incidence at both time points, would have resulted in an increase by slightly more than 0.25 percentage points. The total change, thus, sums up to a rise of lifetime risk by almost 3 percentage points.

## Discussion and conclusion

In this paper, we presented a decomposition method, which allows to separate the contribution of changing incidence and changing survival on the difference between two lifetime risks. The method can be applied to compare lifetime risks over time or between different populations. We illustrated the method and the interpretation of its estimates using hypothetical as well as empirical data for three different diseases, which differ in their development of lifetime risk over time.

In the analysis of time trends of the incidence of a certain disease, lifetime risk may be a suitable summary measure across age categories or lifespan. In such an application, it is conceivable that the incidence decreases with time in parallel with a reduction in death risks, which in turn determines survival. This is the situation in which the change in incidence forces lifetime risk to go down, while the change in survival forces lifetime risk to go up. Thus, the two forces work in opposite directions and a positive development of the risk of getting a disease may be hidden but can be excavated by a decomposition method. Of course, other approaches such as age-standardization are conceivable to investigate the change in incidence but such methods would ignore the quantification of the influence of improved survival. In this context, lifetime risk provides a valuable, perhaps unique, opportunity to combine information on survival and incidence within one single index. The presented decomposition method allows to investigate incidence change and additionally survival change at the same time by comparing directly the observed age-specific incidence and death rates, thereby omitting the use of some arbitrary standard population.

As we have seen from the empirical examples, whether increasing longevity influences the development of lifetime risk or not, depends on the timing of the respective disease. In recent decades, mortality improvements have been especially prevalent at higher ages [16]. Accordingly, especially lifetime risks of diseases, which tend to occur at higher ages, are influenced by increasing survival. This is because improved survival causes a higher number of survivors to the ages where disease incidence is highest and, hence, even when incidence is going down, the declines at those ages are at least compensated by the higher number of persons under risk. In turn, for disease which tend to occur at ages with only marginal survival improvements, incidence declines of the same magnitude would have a stronger effect on lifetime risk. That is because of the number of survivors at that ages, which differs only marginally between the two populations.

Furthermore, it is worth noting that for fatal diseases there is a certain degree of dependency between the disease risks and the death risks. However, as we show in the appendix the method could easily be extended to capture this dependency. In this case, it is a three factor decomposition, which separates, besides the contributions of survival and incidence, the contribution of changes in disease related mortality to incorporate the interrelation of declining incidence as a potential driver of increasing longevity over time. In fact, the approach could even be extended to a four factor decomposition, which could additionally provide the contribution of changing case fatality.

Many common summary measures of population health and mortality are distorted by the increase in longevity, or more generally by the change of the age-structure of the underlying population. The general procedure presented here can add to our understanding of the influences of these factors on such measures. Practically, these decompositions could also be important in forecasting and planning health care resources in the future.

## Supporting information

S1 Appendix Mathematical derivation of the method: Omitted steps. (PDF)

S2 Appendix Extending the decomposition by the disease related mortality rate: Equations and illustrative example. (PDF)
