# Correction: The effects of increasing longevity and changing incidence on lifetime risk differentials: A decomposition approach

**DOI:** 10.1371/journal.pone.0205550

**Published:** 2018-10-10

**Authors:** Marcus Ebeling, Karin Modig, Anders Ahlbom, Roland Rau

There are errors in the second and third sentences of the second paragraph of the Methods, as well as errors in the second and third equations. Please see the corrected sentences and equations here:

Hence, the fraction alive and healthy at age *x*_*i*_ can be calculated by $exp[-\sum_{x\leq y<x_{i}}\mu_{y}]$. The lifetime risk of becoming diseased from age *x* onward, *lr*_*x*_, can then be calculated by

$${lr}_{x}=\sum_{x\leq x_{i}\leq \omega }I_{x_{i}}\exp\left[-\sum_{x\leq y<x_{i}}\mu_{y}\right],$$ (2)

where *ω* denotes the highest age attained. Eq 2 can be rewritten as

$${lr}_{x}=\sum_{x\leq x_{i}\leq \omega }I_{x_{i}}\exp\left[-\sum_{x\leq y<x_{i}}I_{y}\right]\exp\left[-\sum_{x\leq y<x_{i}}m_{y}\right].$$ (3)

For simplicity, we will write $\phi_{x_{i}}$ for $I_{x_{i}}\exp\left[-\sum_{x\leq y<x_{i}}I_{y}\right]$ and $l_{x_{i}}$ for $\exp\left[-\sum_{x\leq y<x_{i}}m_{y}\right]$.

The following changes occurred after the adjustment for the incorrect summation index:

- Myocardial infarction: slight increase in the lifetime risk estimates
- Colorectal cancer: slight increase in the lifetime risk estimates, the total change between the two lifetime risks compared, and the contributions of changes in disease incidence
- Hip fracture: slight increase in the lifetime risk estimates, the total change in the two lifetime risks compared, and the contribution of changes in survival; slight decline in the contribution of changes in disease incidence

These changes are represented in an updated Fig 1 and do not require any updates to the text. Please see the corrected Fig 1 here.

**Fig 1:**
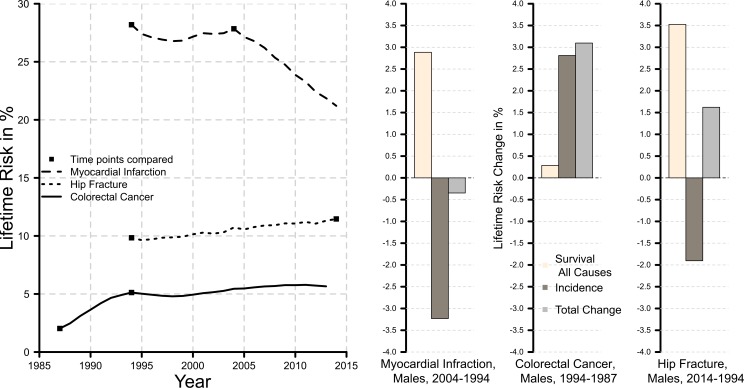
Remaining lifetime risk at age 60 and lifetime risk decomposition for myocardial infarction, hip fracture and colorectal cancer, Sweden, males.

The incorrect summation index also appears in the S2 appendix. Therefore, there are errors in the S2 Appendix. The correct S2 Appendix can be viewed below.

## Supporting information

S2 Appendix Extending the decomposition by the disease related mortality rate: Equations and illustrative example. (PDF)
