# Supplementary material for: The effects of increasing longevity and changing incidence on lifetime risk differentials: A decomposition approach
Source: PLoS One. 2018 Apr 19;13(4):e0195307. doi: 10.1371/journal.pone.0195307 (PMC5909551; doi:10.1371/journal.pone.0195307)
Supplement: S1 Appendix — (PDF) [file pone.0195307.s001.pdf]

The effects of increasing longevity and changing incidence on lifetime risk differentials: A decomposition approach
(Appendix 1)

Marcus Ebeling[*], Karin Modig[†], Anders Ahlbom[‡] and Roland Rau[§]

Mathematical derivation of the method: omitted steps

In the main text, we have not shown the full derivation of the decomposition method. The steps to derive the final decomposition formula are shown below. Starting from the equation

$$\Delta = \sum_{x \le x_i \le \omega} \phi_{x_i,A} l_{x_i,A} - \sum_{x \le x_i \le \omega} \phi_{x_i,B} l_{x_i,B}. \tag{1}$$

we split the two components in the first line of Eq 2 in halves, and add and subtract identical terms in the second line of Eq 2. We do this in order to end up with easily interpretable terms.

$$\begin{aligned}\Delta = &\frac{\sum_{x \le x_i \le \omega} l_{x_i,A}\phi_{x_i,A}}{2} + \frac{\sum_{x \le x_i \le \omega} l_{x_i,A}\phi_{x_i,A}}{2} - \frac{\sum_{x \le x_i \le \omega} l_{x_i,B}\phi_{x_i,B}}{2} - \frac{\sum_{x \le x_i \le \omega} l_{x_i,B}\phi_{x_i,B}}{2} \\ &+ \frac{\sum_{x \le x_i \le \omega} l_{x_i,A}\phi_{x_i,B}}{2} - \frac{\sum_{x \le x_i \le \omega} l_{x_i,A}\phi_{x_i,B}}{2} + \frac{\sum_{x \le x_i \le \omega} l_{x_i,B}\phi_{x_i,A}}{2} - \frac{\sum_{x \le x_i \le \omega} l_{x_i,B}\phi_{x_i,A}}{2}.\end{aligned} \tag{2}$$

By grouping, we can reduce Eq 2 to four terms:

$$\begin{aligned}\Delta = &\sum_{x \le x_i \le \omega} l_{x_i,A} \frac{\phi_{x_i,A} + \phi_{x_i,B}}{2} + \sum_{x \le x_i \le \omega} \phi_{x_i,A} \frac{l_{x_i,A} + l_{x_i,B}}{2} \\ &- \sum_{x \le x_i \le \omega} l_{x_i,B} \frac{\phi_{x_i,A} + \phi_{x_i,B}}{2} - \sum_{x \le x_i \le \omega} \phi_{x_i,B} \frac{l_{x_i,A} + l_{x_i,B}}{2}.\end{aligned} \tag{3}$$

[*]University of Rostock & Max Planck Institute for Demographic Research, Rostock, Germany
[†]Karolinska Institutet, Stockholm, Sweden
[‡]Karolinska Institutet, Stockholm, Sweden
[§]University of Rostock & Max Planck Institute for Demographic Research, Rostock, Germany

Eq 3 can now be rewritten to get the decomposition formula

$$\Delta = \underbrace{\sum_{x \le x_i \le \omega} \left[l_{x_i,A} - l_{x_i,B} \right] \frac{\phi_{x_i,A} + \phi_{x_i,B}}{2}}_{\text{Contribution of Changing Survival Conditions}} + \underbrace{\sum_{x \le x_i \le \omega} \left[\phi_{x_i,A} - \phi_{x_i,B} \right] \frac{l_{x_i,A} + l_{x_i,B}}{2}}_{\text{Contribution of Changes in Incidence}} . \tag{4}$$
