# Supplementary material for: Correction: The effects of increasing longevity and changing incidence on lifetime risk differentials: A decomposition approach
Source: PLoS One. 2018 Oct 10;13(10):e0205550. doi: 10.1371/journal.pone.0205550 (PMC6179262; doi:10.1371/journal.pone.0205550)
Supplement: S2 Appendix — (PDF) [file pone.0205550.s001.pdf]

The effects of increasing longevity and changing incidence on lifetime risk differentials: A decomposition approach (Appendix 2)

Marcus Ebeling[*], Karin Modig[†], Anders Ahlbom[‡] and Roland Rau[§]

Extending the decomposition by the disease related mortality rate: equations and illustrative example

To investigate and illustrate the relationship between declining incidence of the respective disease and its role as a potential driver of increasing longevity over time, we incorporate disease-related mortality as a third factor to the decomposition. If we are including the death rate of the respective disease at age x, d_x, the rate of either dying or getting diagnosed at age x changes to

$$\mu_x = m_x + I_x + d_x, \tag{1}$$

where, for interpretation reasons, m_x now depicts the death rate for all other causes than the disease in question. By applying this modification, the calculation of lifetime risk changes to

$$lr_x = \sum_{x \le x_i \le \omega} I_{x_i}\, exp[-\sum_{x \le y < x_i} I_y]\, exp[-\sum_{x \le y < x_i} m_y]\, exp[-\sum_{x \le y < x_i} d_y]. \tag{2}$$

For simplicity, we will write again ϕ_{x_i} for $I_{x_i}\, exp[-\sum_{x \le y < x_i} I_y]$ and l_{x_i} for $exp[-\sum_{x \le y < x_i} m_y]$ and, furthermore, $\lambda_{x,i}$ for $exp[-\sum_{x \le y < x_i} d_y]$. Given these changes, the original decomposition formula is extended by a third term, which indicates the contribution of changes in disease-specific

[*]University of Rostock & Max Planck Institute for Demographic Research, Rostock, Germany
[†]Karolinska Institutet, Stockholm, Sweden
[‡]Karolinska Institutet, Stockholm, Sweden
[§]University of Rostock & Max Planck Institute for Demographic Research, Rostock, Germany

mortality between both populations. Accordingly, the formula writes as follows

$$
\begin{aligned}
&\sum_{x\le x_i\le\omega}\phi_{x_i,A}l_{x_i,A}\lambda_{x_i,A}-\sum_{x\le x_i\le\omega}\phi_{x_i,B}l_{x_i,B}\lambda_{x_i,B}= \\
&\underbrace{\sum_{x\le x_i\le\omega}(\phi_{x_i,A}-\phi_{x_i,B})\left[\frac{l_{x_i,A}\lambda_{x_i,A}+l_{x_i,B}\lambda_{x_i,B}}{3}+\frac{l_{x_i,A}\lambda_{x_i,B}+l_{x_i,B}\lambda_{x_i,A}}{6}\right]}_{\text{Contribution of Changing Incidence Risks}} \\
&+\underbrace{\sum_{x\le x_i\le\omega}(l_{x_i,A}-l_{x_i,B})\left[\frac{\phi_{x_i,A}\lambda_{x_i,A}+\phi_{x_i,B}\lambda_{x_i,B}}{3}+\frac{\phi_{x_i,A}\lambda_{x_i,B}+\phi_{x_i,B}\lambda_{x_i,A}}{6}\right]}_{\text{Contribution of Changing Survival Conditions (except Disease)}} \\
&+\underbrace{\sum_{x\le x_i\le\omega}(\lambda_{x_i,A}-\lambda_{x_i,B})\left[\frac{\phi_{x_i,A}l_{x_i,A}+\phi_{x_i,B}l_{x_i,B}}{3}+\frac{\phi_{x_i,A}l_{x_i,B}+\phi_{x_i,B}l_{x_i,A}}{6}\right]}_{\text{Contribution of Changing Disease Related Survival}}
\end{aligned}
\tag{3}
$$

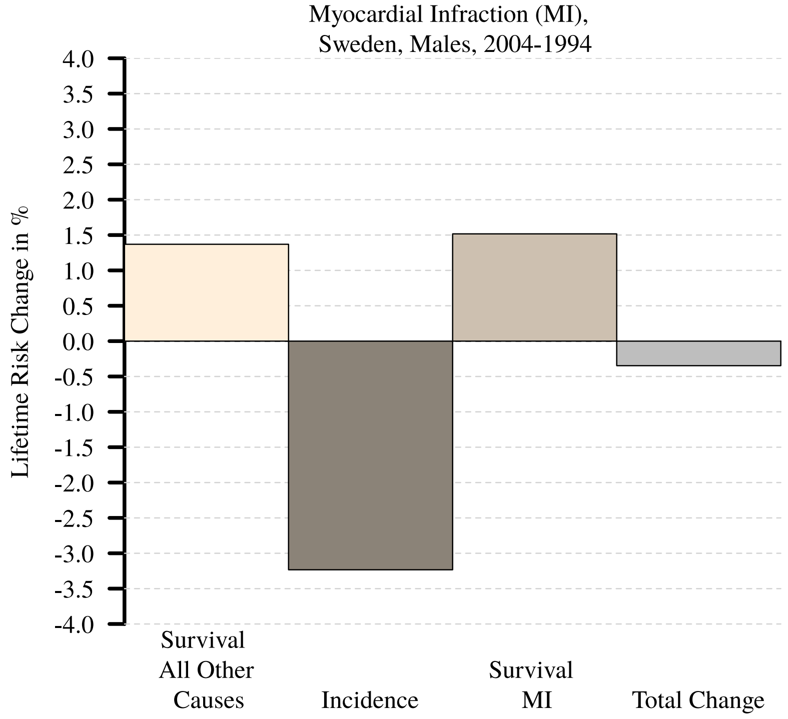

Figure 1: **Decomposition of Lifetime Risk at Age 60 for Myocardial Infarction, Sweden, Males, 1994-2004.**

Fig. 1 shows an application of the three factor decomposition to the example of lifetime risk for myocardial infarction for Swedish males, comparing the years 1994 and 2004. During

this period, lifetime risk almost stagnated, which could lead to the presumption that there has been no improvement in the incidence of getting a myocardial infarction. By applying the three factor decomposition, however, we see that the opposite is true. Not only incidence declined, also the mortality of myocardial infarction improved. Given the same mortality for myocardial infarction and all other causes, the contribution of changing incidence between both time points would have resulted in a decrease of lifetime risk by more than 3 percentage points. However, the improved survival for both myocardial infarction and all other causes prevented this decline. Hence, given the same incidences and mortality of myocardial infarction at both time points, the contribution of increased survival for all other causes would have resulted in a rise of lifetime risk by more than 1.25 percentage points. Additionally, improved mortality for myocardial infarction would have resulted in an increase by almost 1.5 percentage points. In sum, the three factors add up to a total change of less than -0.5 percentage points.
